# Stigma Experiences of Sexual and Gender Minority Parents and Offspring Mental Health

**DOI:** 10.1001/jamanetworkopen.2025.4502

**Published:** 2025-04-10

**Authors:** Qimin Liu, Mingcong Tang, Violeta J. Rodriguez

**Affiliations:** 1 Department of Psychological and Brain Sciences, Boston University, Boston, Massachusetts; 2 Department of Psychology, University of Illinois Urbana-Champaign

## Abstract

**Importance:**

Nearly 40% of sexual and gender minority individuals become parents. Research has highlighted the intergenerational outcomes of parental psychopathology associated with child psychiatric symptoms, yet how stigma and parental mental health influence child outcomes in sexual and gender minority families remains unclear.

**Objective:**

To examine associations between parental stigma experiences and psychiatric symptoms and children’s mental health and emotional and behavioral well-being.

**Design, Setting, and Participants:**

This survey study recruited a community-based sample of sexual and gender minority parents (aged ≥18 years) between October 12 and December 1, 2023. Parents reported stigma experiences, internalizing and externalizing psychopathology, and their children’s emotional and conduct problems.

**Exposure:**

Parental stigma defined as discrimination and internalized stigma.

**Main Outcomes and Measures:**

Parental externalizing and internalizing psychopathology and child emotional and conduct problems were analyzed using structural equation modeling with bifactor measurement models.

**Results:**

The sample included 551 sexual and gender minority parents (mean [SD] age, 34.5 [8.7] years, 268 identifying as cisgender women [48.6%]). Parental psychiatric symptoms were significantly associated with children’s psychiatric symptoms (β [SE], 9.35 [3.44]; 95% CI, 2.61-16.09). Parental externalizing symptoms were associated with child conduct problems (β [SE], 0.67 [0.32]; 95% CI, 0.03-1.30), while internalizing symptoms were associated with child emotional problems (β [SE], 2.05 [0.77]; 95% CI, 0.54-3.55). General stigma was associated with both child psychiatric symptoms (β [SE], 3.53 [1.20]; 95% CI, 1.18-5.89) and emotional problems (β [SE], 2.13 [0.45]; 95% CI, 1.25-3.01). Discrimination, was also significantly associated with child emotional problems (β [SE], 0.22 [0.11]; 95% CI, 0.00-0.44).

**Conclusions and Relevance:**

This survey study found that parental stigma experiences in sexual and gender minority families are associated with both parental and child psychopathology. These findings highlight the need for longitudinal, multi-informant research to guide interventions supporting sexual and gender minority family mental health.

## Key Points

**Question:**

Are parents’ stigma experiences and psychiatric symptoms associated with their children’s psychiatric symptoms in sexual and gender minority families?

**Findings:**

In this survey study of 551 sexual and gender minority parents, parental externalizing symptoms were associated with child conduct problems, and parental internalizing symptoms were associated with child emotional problems. General parental stigma was associated with both children’s overall psychopathology and emotional problems specifically, as well as parents’ internalizing psychopathology, while parents’ discrimination was associated with child emotional problems.

**Meaning:**

These findings underscore the need for further longitudinal and multi-informant research to guide interventions that support sexual and gender minority family mental health.

## Teaser

This survey study examined the associations of sexual and gender minority parental stigma experiences and psychiatric symptoms with their children’s mental health and emotional and behavioral well-being.

## Introduction

Approximately 37% of sexual and gender minority adults have children,^1^ yet research on their mental health needs remains limited. Sexual and gender minority families face unique stressors, including societal stigma and systemic discrimination, which may be factors associated with child mental health. However, most studies have focused on heteronormative families, leaving a gap in understanding these dynamics in sexual and gender minority families.

Studies have suggested that parental mental health transmits across generations.^2,3^ Internalizing (eg, anxiety, depression) and externalizing (eg, anger, aggression) symptoms among sexual and gender minority parents may influence children’s emotional and behavioral well-being.^4^ However, sexual and gender minority families face compounded stressors due to their marginalized identities, making existing findings potentially incomplete.

Sexual and gender minority parents experience stigma at multiple levels, including societal, systemic, and interpersonal, which may exacerbate stress and psychiatric symptoms.^5^ Minority stress theory posits that marginalization negatively impacts mental health.^6^ While extensively applied to adults, its effects on children in sexual and gender minority families remain understudied. Research on stigma’s role in intergenerational transmission of psychiatric symptoms is scarce,^7,8,9^ as the harms of discrimination may be transmitted across generations.^10^ Stigma may not only harm parents but also affect their children’s well-being.^5,11,12^ Yet, few studies have examined how parental stigma is associated with child mental health, limiting intervention efforts. Consequently, the potential to support the well-being of children with sexual and gender minority parents is considerably hindered.

The conceptual model in this study is grounded in established theoretical frameworks, particularly the intergenerational transmission of psychopathology^13,14^ and minority stress theory.^5^ These frameworks emphasize the influence of parental mental health and stigma experiences on child mental health outcomes. Prior research has supported the multidimensional nature of stigma and psychopathology, necessitating an analytic approach that accounts for both general and specific factors.^4,15^ Existing research on sexual and gender minority families is also limited by a predominant focus on White, socioeconomically advantaged, primarily sexual minority individuals, failing to represent the diversity of the sexual and gender minority community and restricting the generalizability of findings. This narrow scope of previous research overlooks the unique experiences and challenges faced by a substantial portion of the sexual and gender minority population. To address this limitation, it is essential to broaden the scope of research to include ethnoracially and socioeconomically diverse sexual and gender minority families.

In this study, we examined whether and how parental experiences of stigma and psychiatric symptoms are associated with the mental health outcomes of children in sexual and gender minority families. Specifically, we hypothesized that (1) parental internalizing symptoms may be positively associated with child emotional problems, (2) parental externalizing symptoms may be positively associated with child conduct problems, and (3) parental experiences of stigma may be associated with both parental psychiatric symptoms and child mental health outcomes.

## Methods

### Participants

For this survey study, we recruited sexual and gender minority parents aged 18 years or older with at least 1 child aged 3 to 17 years and living with the child at least 50% of the time. If a parent had more than 1 child, they were asked to select the child with the most recent birthday for the survey. To ensure data independence, only 1 parent per family could complete the surveys. An additional eligibility criterion required parents to identify as members of the lesbian, gay, bisexual, transgender, queer (or questioning), asexual (or allied), intersex, or other sexual or gender identity community. Parents were not informed of the eligibility criteria but were not included after screening if they did not meet the requirements based on a preliminary survey used to determine eligibility. Study materials and procedures were approved by the institutional review boards of Boston University and the University of Illinois Urbana-Champaign. Participants were presented with an online consent form outlining the study’s purpose, procedures, potential risks and benefits, and their rights as participants. Because no personally identifiable information was collected, consent was provided anonymously. Participants indicated their agreement before proceeding with the survey. No names or identifying details were recorded, ensuring confidentiality in participation. We followed the American Association for Public Opinion Research (AAPOR) reporting guideline.

The survey was distributed nationwide through Qualtrics. Qualtrics assessed participant eligibility from its prescreened panels and emailed the survey to eligible participants with a unique link, subsequently disbursing compensation as an incentive. Recruitment was completed from October 12 to December 31, 2023. Participants who correctly answered attention and quality checks embedded in the survey were included in the final dataset.

### Measures

Participants were asked to complete a sociodemographic questionnaire about their age, gender identity (cisgender man, cisgender woman, gender nonbinary, transgender man, transgender woman), race (American Indian or Alaska Native, Asian, Black or African American, White, or other [reported by a substantial number of participants as their nationality, eg, Greek, Mexican, Romanian, or their ethnicity, eg, Latinx), ethnicity (Hispanic or non-Hispanic), sexual orientation (bisexual, gay/lesbian, heterosexual, other orientations), educational attainment, and household income. Parenting-related questions included the gender and age of the child. Participants were provided with a list of options for gender identity, race and ethnicity, and sexual orientation. This method was chosen to maintain consistency, reduce variability in responses, and align with established demographic categorization practices. Race and ethnicity referenced US Census Bureau categories, whereas sexual and gender minority categories referenced American Psychological Association guidelines.

### Parental Stigma Experiences

The Everyday Discrimination Scale^16^ was used to assess perceived everyday discrimination experienced by parents in their daily lives. This scale evaluates the frequency of 9 types of everyday discrimination events on a 6-point scale ranging from 1 (never) to 6 (almost every day). The Everyday Discrimination Scale has demonstrated good reliability, with a coefficient ω of 0.94 in the current study.

The Internalized Sexual Stigma Scale is a 7-item assessment adapted from the Internalized Homophobia Scale^17^ to assess the internalization of negative societal attitudes toward one’s sexual orientation. The Internalized Sexual Stigma Scale uses a 7-point Likert scale ranging from 1 (strongly disagree) to 7 (strongly agree), with higher scores indicating greater levels of internalized sexual stigma. The Internalized Sexual Stigma Scale has demonstrated good reliability, with a coefficient ω of 0.96 in the current study.

The Internalized Transphobia Subscale from the Gender Minority Stress and Resilience Measure^18^ was used to measure internalized transphobia. The subscale consists of 6 items, each on a 4-point Likert scale ranging from 1 (strongly disagree) to 4 (strongly agree), with higher scores indicating greater levels of internalized transphobia. The Internalized Transphobia Subscale has demonstrated good reliability, with a coefficient ω of 0.93 in the current study.

### Parental Psychiatric Symptoms

We assessed internalizing symptoms, including symptoms of depression and anxiety. We also assessed irritable mood and aggressive outbursts as measures of externalizing symptoms.

The Patient Health Questionnaire-9 was used to assess parents’ depressive symptoms.^19^ It consists of 9 items that assess the presence and frequency of depressive symptoms over the past 2 weeks. Each item is scored on a 4-point scale ranging from 0 (not at all) to 3 (nearly every day). The total score ranges from 0 to 27, with higher scores indicating more severe depression. The Patient Health Questionnaire-9 has demonstrated good reliability, with a coefficient ω of 0.94 in the current study.

The Generalized Anxiety Disorder-7^20^ instrument was used to assess parents’ anxiety symptoms. It consists of 7 items that assess the presence and frequency of generalized anxiety symptoms over the past 2 weeks. Each item is scored on a 4-point scale ranging from 0 (not at all) to 3 (nearly every day). The total score ranges from 0 to 21, with higher scores indicating more severe anxiety. The Generalized Anxiety Disorder-7 has demonstrated good reliability, with a coefficient ω of 0.94 in the current study.

The Brief Irritability Test is a 5-item questionnaire^21^ used to measure irritability, which is characterized by a persistently angry, grumpy, or bad-tempered mood. The questionnaire items are measured on a 6-point scale ranging from never to always. Participants are asked to indicate how often they have felt or behaved in certain ways during the past 2 weeks, including the day of the test. The current study used only the original items 1 through 4 of the Brief Irritability Test questionnaire. The fifth item, “Things have been bothering me more than they normally do,” was not included in the measurement because we considered this item to be nonspecific and related to general distress rather than as a specific assessment of externalizing symptoms. The Brief Irritability Test has demonstrated good reliability, with a coefficient ω of 0.92 in the current study.

We also included questions related to aggressive outbursts based on the World Health Organization’s World Mental Health Composite International Diagnostic Interview.^22^ Items assessed aggressive outbursts, including physical aggression, verbal aggression, and property destruction. Items were rated on a 6-point scale ranging from 1 (never) to 6 (always), with higher scores indicating higher externalizing symptoms. The questions have demonstrated good reliability, with a coefficient ω of 0.90 in the current study.

### Children’s Emotional and Conduct Problems

The Strengths and Difficulties Questionnaire is a brief behavioral screening questionnaire for children and adolescents aged 2 to 17 years.^23^ It consists of 25 items divided into 5 subscales: emotional symptoms, conduct problems, hyperactivity/inattention, peer relationship problems, and prosocial behavior. Each item is scored on a 3-point scale of 0 (not true), 1 (somewhat true), and 2 (certainly true). Child psychopathology is the sum of scores of emotional symptoms, conduct problems, hyperactivity/inattention, and peer relationship problems, with higher scores indicating greater symptoms. The questionnaire items vary slightly according to child’s age group.

### Statistical Analysis

A bifactor structural equation modeling approach was used to capture both shared and unique variance within latent constructs, such as parental stigma and internalizing and externalizing symptoms. This model allows for the estimation of a general factor (eg, overall stigma, psychopathology) alongside domain-specific factors (eg, discrimination, internalized stigma, depression, anxiety), improving interpretability and minimizing multicollinearity.^15^ Bifactor structural equation modeling has been effectively applied in prior studies to disentangle complex constructs in stigma and mental health research.^15,24^ In sum, we used a structural equation model to examine the associations among parental stigma, parental psychiatric symptoms, and child psychiatric symptoms.

The model includes measurement components that disaggregate general from specific factors of parental stigma and psychiatric symptoms. Parental general experiences of stigma were indicated by items of everyday discrimination, internalized sexual stigma, and internalized transphobia. A specific factor of enacted stigma was indicated by items of everyday discrimination, whereas a specific factor of felt stigma was indicated by items of internalized sexual stigma and internalized transphobia. Parental general internalizing symptoms were indicated by items of anxiety and depression, whereas items specific to anxiety and to depression comprised the specific factors for anxiety and for depression, respectively. Similarly, parental general externalizing symptoms were indicated by items of irritable mood and aggressive outbursts, whereas items specific to irritable mood and to aggressive outbursts consisted of the specific factors for irritable mood and for aggressive outbursts, respectively. We constructed an additional factor of parental overall psychiatric symptoms indicated by latent general factors of internalizing and externalizing symptoms with fixed unit weights. The structural components of the model tested the associations from general and specific factors of parental stigma and psychiatric symptoms to child emotional and conduct problems and overall mental health (Figure).

**Figure.**
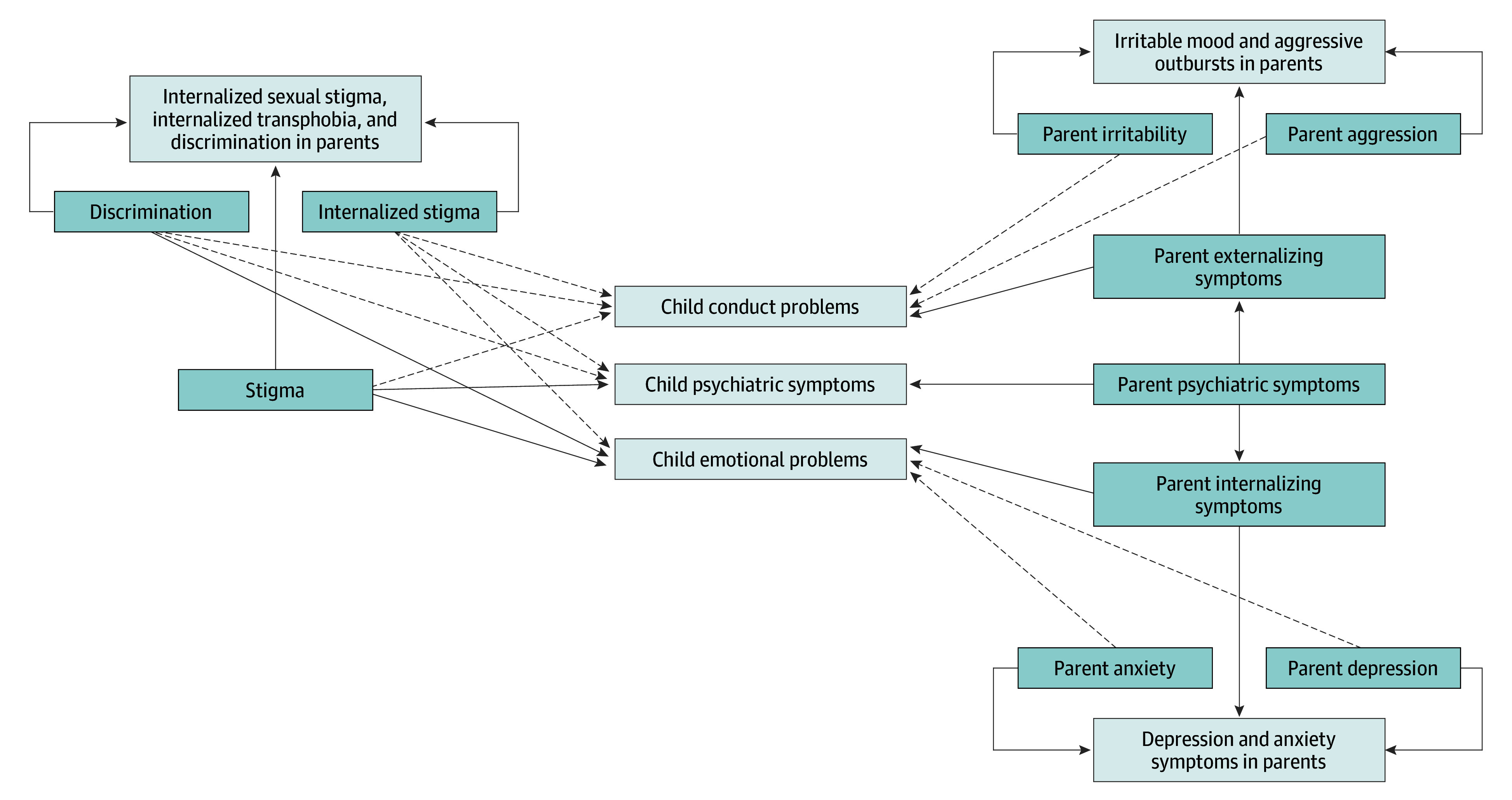
Structural Equation Model of Stigma Experiences and Parental and Child Psychiatry Symptoms The solid lines reflect the structural model connecting parental stigma and psychopathology to child psychopathology (significant). The dashed lines reflect the pathways that were not significant. Boxes with darker shading denote latent variables, whereas boxes with lighter shading denote observed variables.

The adequacy of the sample size in bifactor structural equation modeling depends on model complexity, data quality, and estimation methods. Research has suggested a minimum of 200 to 300 participants for complex structural equation models and 300 to 500 for bifactor models.^15,25,26^ Our sample size exceeded these thresholds, supporting robust estimation. The model converged without issues, and fit indices (comparative fit index, 0.922; Tucker-Lewis index, 0.912, root mean square error of approximation, 0.054; standardized root mean squared residual, 0.043) indicated good model fit.^27^ The statistical analyses were conducted using R, version 4.3.1 (R Foundation). We used bifactor models to examine the factor structure, implemented through the R package lavaan, version 0.6-19. The threshold for statistical significance was set at *P* < .05.

## Results

The study included a total of 551 sexual and gender minority parents (mean [SD] age, 34.5 [8.7] years). Most participants identified as bisexual (290 [52.6%] vs 90 gay or lesbian [16.4%], 51 heterosexual [9.3], and 120 another sexual orientation [21.8%]), cisgender women (268 [48.6%] vs 72 identifying as cisgender men [13.1%], 190 as gender nonbinary [34.5%], 12 as transgender men [2.2%], and 9 as transgender women [1.6%]), and Hispanic or Latino (157 [28.0%] vs 94 identifying as American Indian or Alaska Native [17.0%], 82 as Asian [15.0%], 86 as Black [16.0%], 49 as White [8.9%], and 83 as other [15.0%] race and ethnicity). Children’s mean (SD) age was 10.2 (4.5) years, with the majority identified as gender nonbinary (204 [37.0%] vs 168 identified as cisgender boys [31.0%], 152 as cisgender girls [28.0%], and 19 as transgender boy or girl [3.5%]). Detailed participant demographics are presented in Table 1.

**Table 1.** Child and Parent Demographic Characteristics (N = 551)

Characteristic	Participants, No. (%)
**Child**	
Age, mean (SD), y	10.2 (4.5)
Gender identity	
Cisgender boy	168 (31.0)
Cisgender girl	152 (28.0)
Gender nonbinary	204 (37.0)
Transgender boy	11 (2.0)
Transgender girl	8 (1.5)
Other^a^	2 (1.5)
**Parent**	
Age, mean (SD), y	34.5 (8.7)
Sexual orientation	
Bisexual	290 (52.6)
Gay or lesbian	90 (16.4)
Heterosexual	51 (9.3)
Other^b^	120 (21.8)
Gender identity	
Cisgender man	72 (13.1)
Cisgender woman	268 (48.6)
Gender nonbinary	190 (34.5)
Transgender man	12 (2.2)
Transgender woman	9 (1.6)
Race	
American Indian or Alaska Native	94 (17.0)
Asian	82 (15.0)
Black or African American	86 (16.0)
Hispanic	157 (28.0)
White	49 (8.9)
Other^c^	83 (15.0)
Ethnicity	
Hispanic or Latino	172 (31.2)
Non-Hispanic	379 (68.8)
Education	
High school or less	174 (31.6)
Associate’s degree	69 (13.0)
Some college	135 (25.0)
Bachelor’s degree	116 (21.0)
Postgraduate degree^d^	57 (10.0)
Annual household income, $	
<10 000	63 (11.0)
10 001-30 000	101 (18.0)
30 001-50 000	114 (21.0)
50 001-70 000	109 (20.0)
70 001-90 000	60 (11.0)
90 001-110 000	46 (8.3)
>110 000	44 (8.0)
Preferred not to answer	14 (2.5)

^a^ Including, for example, demigender and gender fluid.

^b^ Including, for example, demisexual and polysexual.

^c^ A substantial number of participants reported their nationality (eg, Greek, Mexican, Romanian) or instead of race, reported their ethnicity (eg, Latinx).

^d^ Included master’s degree, specialist, PhD, MD, and JD.

### Preliminary Checks

The structural equation model used maximum likelihood estimation,^28^ which provides unbiased parameter estimates and is commonly used in structural equation modeling.^26^ Normality was assessed via skewness and kurtosis, with no severe deviations detected.^26^ Multicollinearity was examined using variance inflation factor and correlation analysis, with all variance inflation factor values less than 5 and no correlations exceeding 0.85, indicating no major concerns^29^ (eTables 2 and 3 in Supplement 1). To handle missing data (20.4%), the full information maximum likelihood algorithm in lavaan was used, allowing for unbiased estimation by using all available data without imputation. Full information maximum likelihood retains the full sample and is more efficient than traditional deletion methods.^28^ It is widely recognized for its accuracy and robustness in structural equation modeling, including bifactor models, even with moderate missing data levels.^30^

### Child Mental Health

Parental general psychiatric symptoms (β [SE], 9.35 [3.44]; 95% CI, 2.61-16.09; *z* score, 2.72; *P* = .007) and parental general stigma (β [SE], 3.53 [1.20]; 95% CI, 1.18-5.89; *z* score, 2.94; *P* = .003) were significantly associated with overall child psychiatric symptoms (Table 2). Parental enacted stigma (ie, discrimination) (β [SE], 0.55 [0.41]; 95% CI, −0.25 to 1.35; *z* score, 1.34; *P* = .18) or felt stigma (β [SE], 0.25 [0.39]; 95% CI, −0.52 to 1.03; *z* score, 0.64; *P* = .52) was not significantly associated with overall child psychiatric symptoms after accounting for the general parental stigma.

**Table 2.** Regression Pathways of Stigma Experiences and Parental Psychiatric Symptoms Associated With Child Psychiatric Symptoms^a^

Child outcome	β (SE) [95% CI]^b^	*z* Score	*P* value	Standard estimate
**Mental health**				
Parental psychiatric symptoms	9.35 (3.44) [2.61 to 16.09]	2.72	.007	0.45
Stigma	3.53 (1.20) [1.18 to 5.89]	2.94	.003	0.22
Discrimination	0.55 (0.41) [–0.25 to 1.35]	1.34	.18	0.09
Internalized stigma	0.25 (0.39) [–0.52 to 1.03]	0.64	.52	0.03
**Conduct problems**				
Parental externalizing symptoms				
General	0.67 (0.32) [0.03 to 1.30]	2.05	.04	0.18
Irritability	−3.03 (6.01) [–14.81 to 8.75]	−0.51	.61	−1.12
Aggression	−6.18 (12.83) [–31.33 to 18.98]	−0.48	.63	−1.70
Stigma	9.23 (17.29) [–24.65 to 43.11]	0.53	.59	1.97
Discrimination	1.77 (3.38) [–4.86 to 8.40]	0.52	.60	1.00
Internalized stigma	1.04 (2.33) [–3.52 to 5.60]	0.45	.66	0.43
**Emotional problems**				
Parental internalizing symptoms				
General	2.05 (0.77) [0.54 to 3.55]	2.66	.008	0.28
Depression	0.08 (0.26) [–0.44 to 0.59]	0.29	.77	0.01
Anxiety	0.24 (0.21) [–0.18 to 0.66]	1.13	.26	0.07
Stigma	2.13 (0.45) [1.25 to 3.01]	4.73	<.001	0.34
Discrimination	0.22 (0.11) [0.00 to 0.44]	1.98	.05	0.09
Internalized stigma	0.08 (0.17) [–0.26 to 0.42]	0.47	.64	0.03

^a^ Model fit: comparative fit index, 0.922; Tucker-Lewis index, 0.912; root mean square error of approximation, 0.054; standardized root mean square residual, 0.043. The comparative fit index indicates how well the model fits compared with a baseline (null) model, with values of at least 0.90 suggesting acceptable fit and at least 0.95 indicating excellent fit. The comparative fit index value suggested the model had an acceptable fit. The Tucker-Lewis index adjusts for model complexity and measures relative fit, with values of at least 0.90 indicating acceptable fit and at least 0.95 excellent fit. The Tucker-Lewis index value suggested that the model had an acceptable fit, though slightly below excellent standards. The root mean square error of approximation reflects the error of approximation per *df*, with values of 0.06 or less indicating good fit and 0.08 or less indicating acceptable fit. The root mean square error of approximation indicated good model fit. The standardized root mean square residual measures the average discrepancy between observed and estimated correlations, with values of 0.08 or less considered acceptable. The standardized root mean square indicated good model fit.

^b^ Unstandardized estimates.

### Child Conduct Problems

Parental general externalizing symptoms were significantly associated with child conduct problems (β [SE], 0.67 [0.32]; 95% CI, 0.03-1.30; *z* score, 2.05; *P* = .04) (Table 2). Irritable mood (β [SE], −3.03 [6.01]; 95% CI, −14.81 to 8.75; *z* score, −0.51; *P* = .61) and aggressive outbursts (β [SE] = −6.18 [12.83]; 95% CI, −31.3 to 18.98; *z* score, −0.48; *P* = .63) were not significantly associated with child conduct problems, after accounting for parental general externalizing symptoms. Parental general experiences of stigma (β [SE], 9.23 [17.29]; 95% CI, −24.65 to 43.11; *z* score, 0.53; *P* = .59), enacted stigma (β [SE], 1.77 [3.38]; 95% CI, −4.86 to 8.40; *z* score, 0.52; *P* = .60), and felt stigma (β [SE], 1.04 [2.33]; 95% CI, −3.52 to 5.60; *z* score, 0.45; *P* = .66) were also not significantly associated with child conduct problems.

### Child Emotional Problems

Parental general internalizing symptoms were significantly associated with child emotional problems (β [SE], 2.05 [0.77]; 95% CI, 0.54-3.55; *z* score, 2.66; *P* = .008). Parental depression (β [SE], 0.08 [0.26]; 95% CI, −0.44 to 0.59; *z* score, 0.29; *P* = .77) and parental anxiety (β [SE], 0.24 [0.21]; 95% CI, −0.18 to 0.66; *z* score, 1.13; *P* = .26) were not significantly associated with child emotional problems, after accounting for parental general internalizing symptoms. Parental general experiences of stigma were significantly associated with child emotional problems (β [SE], 2.13 [0.45]; 95% CI, 1.25-3.01; *z* score, 4.73; *P* < .001). Enacted stigma was also significantly associated with child emotional problems above and beyond the parental general experiences of stigma (β [SE], 0.22 [0.11]; 95% CI, 0.00-0.44; *z* score, 1.98; *P* = .05). Felt stigma was not significantly associated with child emotional problems (β [SE], 0.08 [0.17]; 95% CI, −0.26 to 0.42; *z* score, 0.47; *P* = .64). Supplementary analyses incorporating age, gender, and race and ethnicity as covariates in the structural equation model are provided in eTable 1 in Supplement 1. Overall, parental psychiatric symptoms and stigma remained consistent factors associated with child psychopathology regardless of adjustments.

## Discussion

This survey study investigated the association of parental stigma and psychiatric symptoms with child mental health in sexual and gender minority families. The findings align with intergenerational transmission theory^7,31^ and minority stress theory,^5^ showing how stress and psychopathology pass across generations in groups that have been historically marginalized.

Consistent with intergenerational transmission theory,^32,33^ parental psychiatric symptoms were significantly associated with children’s symptoms. Internalizing symptoms in parents were associated with children’s internalizing symptoms, while externalizing symptoms were associated with conduct problems. These findings emphasize the importance of family-centered mental health interventions, particularly for groups that have been historically marginalized. Our findings align with research showing the crossover effect of parental mental health on child development,^2,9,34^ reinforcing the need for interventions targeting both parents and children.

Sexual and gender minority families face systemic barriers that exacerbate mental health challenges. Discriminatory policies, socioeconomic disparities, and inadequate access to culturally competent care increase stress and psychopathology transmission. Sexual and gender minority families are often underserved in clinical settings, where stigma and discrimination limit effective support. This lack of tailored care hinders early intervention, reinforcing mental health disparities.

A key assumption in bifactor modeling is the independence of general and specific factors. Research has supported this distinction, as discrimination and internalized stigma function through different mechanisms.^5,6^ Discrimination reflects external prejudice, whereas internalized stigma involves negative self-perceptions. Similarly, bifactor models help differentiate general and specific symptom dimensions in psychopathology research. Enforcing orthogonality clarifies shared vs unique variance in complex constructs.^15,24^ Empirical evidence supports this model’s adequacy (comparative fit index, 0.922; Tucker-Lewis index, 0.912; root mean square error approximation, 0.054; standardized root mean square residual, 0.043).

A notable finding is the significant association between parental stigma and child emotional problems,^35^ reflecting how societal stigma influences child mental health and suggesting that interventions should target broader systemic stigma, not just individual experiences. The association between discrimination and child emotional problems further supports the need for culturally attuned interventions that address family-wide stigma.^35^

Interestingly, neither enacted nor felt stigma were associated with child psychiatric symptoms after accounting for general stigma.^5,6^ This finding suggests that broader, persistent stigma may be more harmful than isolated incidents. Comprehensive antistigma initiatives may be more effective than targeting specific discriminatory events.

Future studies should use longitudinal designs to clarify causal pathways between parental stigma and child mental health.^16,36,37^ Research should also incorporate intersectionality since stigma and psychiatric outcomes vary across race and ethnicity, gender, and socioeconomic status. Multi-informant and mixed-methods approaches may enrich understanding by capturing both statistical patterns and personal narratives. Identifying protective factors, such as social support and family cohesion, is crucial.^5,11,12^ These resilience mechanisms may inform interventions to mitigate outcomes associated with stigma. Future research should also address systemic health disparities by advocating for increased access to inclusive mental health care.

### Limitations

This study has some limitations. Family composition factors (eg, divorce, coparenting) were not accounted for, though they may influence psychiatric symptom transmission. Additionally, children’s direct experiences of stigma were not assessed. Future studies should examine how child stigma experiences shape mental health outcomes independently of parental experiences.

## Conclusions

This survey study found significant associations between parental stigma and psychiatric symptoms and child mental health in sexual and gender minority families. Targeted interventions must focus on both reducing stigma and improving parental mental health. Clinical strategies should help parents manage internalized stigma while fostering community support. Community programs promoting acceptance and antidiscrimination efforts are essential for protecting sexual and gender minority family well-being. Continued research is needed to develop interventions that break the cycle of stigma-related mental health challenges in sexual and gender minority families.

## Notes

Supplement 1. **eTable 1.** Regression Pathways of the Association of Stigma Experience and Parental Psychiatric Symptoms With Child Psychiatric Symptoms With Covariates **eTable 2.** Variance Inflation Factors (VIFs) for Psychometric Items Assessing Stigma, Mental Health, and Gender Measures **eTable 3.** Descriptive Statistics of Psychometric Items: Skewness and Kurtosis Metrics

Supplement 2.
Data Sharing Statement
