# Supplementary material for: Stigma Experiences of Sexual and Gender Minority Parents and Offspring Mental Health
Source: JAMA Netw Open. 2025 Apr 10;8(4):e254502. doi: 10.1001/jamanetworkopen.2025.4502 (PMC11986766; doi:10.1001/jamanetworkopen.2025.4502)
Supplement: Supplement 1. — eTable 1. Regression Pathways of the Association of Stigma Experience and Parental Psychiatric Symptoms With Child Psychiatric Symptoms With Covariates eTable 2. Variance Inflation Factors (VIFs) for Psychometric Items Assessing Stigma, Mental Health, and Gender Measures eTable 3. Descriptive Statistics of Psychometric Items: Skewness and Kurtosis Metrics [file jamanetwopen-e254502-s001.pdf]

Supplemental Online Content

Liu Q, Tang M, Rodriguez VJ. Stigma experiences of sexual and gender minority parents and offspring mental health. *JAMA Netw Open*. 2025;8(4):e254502. doi:10.1001/jamanetworkopen.2025.4502

eTable 1. Regression Pathways of the Association of Stigma Experience and Parental Psychiatric Symptoms With Child Psychiatric Symptoms With Covariates

eTable 2. Variance Inflation Factors (VIFs) for Psychometric Items Assessing Stigma, Mental Health, and Gender Measures

eTable 3. Descriptive Statistics of Psychometric Items: Skewness and Kurtosis Metrics

This supplemental material has been provided by the authors to give readers additional information about their work.

© 2025 Liu Q et al. *JAMA Network Open*.

eTable 1. Regression Pathways of the Association of Stigma Experience and Parental Psychiatric Symptoms With Child Psychiatric Symptoms With Covariates

Variables	Estimate[a]	SE	Z	P	ci.lower	ci.upper	Std.Estimate[b]
			**Child Conduct Problems**				
Parent Externalizing Symptoms	0.581	0.31	1.876	0.061	-0.026	1.188	0.123
Parent Irritability	0.114	0.174	0.654	0.513	-0.227	0.454	0.047
Parent Aggression	0.549	0.118	4.654	0	0.318	0.781	0.368
Stigma	0.661	0.417	1.586	0.113	-0.156	1.478	0.149
Discrimination	0.225	0.14	1.611	0.107	-0.049	0.498	0.127
Internalized Stigma	-0.219	0.133	-1.652	0.099	-0.479	0.041	-0.124
Ethnoracial Group	-0.118	0.228	-0.519	0.604	-0.565	0.328	-0.027
Income	-0.014	0.066	-0.218	0.827	-0.144	0.115	-0.012
Education	0.179	0.086	2.082	0.037	0.01	0.347	0.123
			**Child Emotional Problems**				
Parent Internalizing Symptoms	3.195	1.974	1.619	0.105	-0.673	7.063	0.279
Parent Depression	-0.421	0.395	-1.064	0.287	-1.196	0.354	-0.091
Parent Anxiety	0.71	0.314	2.26	0.024	0.094	1.326	0.251
Stigma	1.561	0.607	2.569	0.01	0.37	2.751	0.26
Discrimination	0.102	0.174	0.585	0.558	-0.239	0.443	0.043
Internalized Stigma	0.181	0.177	1.027	0.304	-0.165	0.528	0.076
Ethnoracial Group	-0.017	0.296	-0.056	0.955	-0.597	0.564	-0.003
Income	0.052	0.085	0.615	0.539	-0.115	0.219	0.034
Education	-0.046	0.111	-0.411	0.681	-0.263	0.172	-0.023
			**Child Psychopathology**				

© 2025 Liu Q et al. *JAMA Network Open*.

Parent Psychiatric Symptoms	16.931	7.986	2.12	0.034	1.278	32.584	0.525
Stigma	1.468	1.576	0.932	0.352	-1.62	4.557	0.099
Discrimination	0.604	0.543	1.112	0.266	-0.46	1.668	0.103
Internalized Stigma	0.279	0.402	0.693	0.489	-0.51	1.067	0.048
Ethnoracial Group	-0.182	0.721	-0.253	0.8	-1.595	1.23	-0.012
Income	0.053	0.209	0.252	0.801	-0.357	0.462	0.014
Education	0.191	0.271	0.703	0.482	-0.341	0.723	0.04

Abbreviations: SE, standard error.

[a] unstandardized estimates. [b] standardized estimates.

Model fit: CFI: 0.904; TLI: 0.892; RMSEA: 0.059; SRMR: 0.056.

© 2025 Liu Q et al. *JAMA Network Open*.

eTable 2. Variance Inflation Factors (VIFs) for Psychometric Items Assessing Stigma, Mental Health, and Gender Measures

Variable	VIF
BITe_1	2.774
BITe_2	3.458
BITe_3	3.424
BITe_4	3.770
BITe_5	3.446
BITe_6	4.075
BITe_7	3.195
PHQ901	2.442
PHQ902	3.002
PHQ903	2.513
PHQ904	2.523
PHQ905	2.312
PHQ906	2.875
PHQ907	2.321
PHQ908	2.205
PHQ909	2.385
GAD701	3.323
GAD702	3.909
GAD703	3.804
GAD704	2.717
GAD705	2.865
GAD706	2.488
GAD707	2.567
EDS_1	3.527
EDS_2	3.986

© 2025 Liu Q et al. *JAMA Network Open*.

EDS_3	2.065
EDS_4	3.078
EDS_5	2.180
EDS_6	2.598
EDS_7	2.601
EDS_8	3.037
EDS_9	2.899
Internalized_Homopho_1	2.370
Internalized_Homopho_2	2.556
Internalized_Homopho_3	3.922
Internalized_Homopho_4	4.787
Internalized_Homopho_5	3.552
Internalized_Homopho_6	4.231
Internalized_Homopho_7	3.498
GMS_1	2.091
GMS_2	3.362
GMS_3	3.286
GMS_4	2.252
GMS_5	3.248
GMS_6	2.896

© 2025 Liu Q et al. *JAMA Network Open*.

eTable 3. Descriptive Statistics of Psychometric Items: Skewness and Kurtosis Metrics

Variable	Skewness	Kurtosis
BITe_1	0.329	2.653
BITe_2	0.476	2.383
BITe_3	0.217	2.169
BITe_4	0.350	2.227
BITe_5	1.125	3.053
BITe_6	1.186	3.191
BITe_7	1.267	3.575
PHQ901	0.566	2.288
PHQ902	0.724	2.496
PHQ903	0.294	1.780
PHQ904	0.277	1.828
PHQ905	0.564	2.026
PHQ906	0.645	2.151
PHQ907	0.662	2.155
PHQ908	1.095	2.977
PHQ909	1.310	3.482
GAD701	0.384	2.028
GAD702	0.431	1.990
GAD703	0.323	1.933
GAD704	0.308	1.905
GAD705	0.679	2.200
GAD706	0.293	1.838
GAD707	0.547	1.931
EDS_1	0.055	1.889
EDS_2	0.196	1.847

© 2025 Liu Q et al. *JAMA Network Open*.

EDS_3	0.673	2.523
EDS_4	0.306	1.931
EDS_5	0.745	2.362
EDS_6	0.724	2.216
EDS_7	0.127	1.912
EDS_8	0.521	2.071
EDS_9	0.730	2.349
Internalized_Homopho_1	0.546	1.945
Internalized_Homopho_2	0.725	2.421
Internalized_Homopho_3	0.893	2.575
Internalized_Homopho_4	0.838	2.533
Internalized_Homopho_5	1.321	3.628
Internalized_Homopho_6	0.968	2.700
Internalized_Homopho_7	0.959	2.788
GMS_1	1.248	3.157
GMS_2	1.348	3.578
GMS_3	0.995	2.644
GMS_4	0.612	1.872
GMS_5	1.145	2.969
GMS_6	1.195	3.101

© 2025 Liu Q et al. *JAMA Network Open*.
