# Supplementary material for: Stigma Experiences of Sexual and Gender Minority Parents and Offspring Mental Health
Source: JAMA Netw Open. 2025 Apr 10;8(4):e254502. doi: 10.1001/jamanetworkopen.2025.4502 (PMC11986766; doi:10.1001/jamanetworkopen.2025.4502)
Supplement: Supplement 2. — Data Sharing Statement [file jamanetwopen-e254502-s002.pdf]

Data Sharing Statement

Liu. Stigma Experiences of Sexual and Gender Minority Parents and Offspring Mental Health. *JAMA Netw Open*. Published April 10, 2025. doi:10.1001/jamanetworkopen.2025.4502

Data
Data available: Yes
Data types: Deidentified participant data
How to access data: The analytic code and additional appendices are available on the study's OSF page found here: https://osf.io/s8xpj/?view_only=4598c7667848414f85bb49ea0eb7ceb6.
When available: With publication

Supporting Documents
Document types: Statistical/analytic code
How to access documents: The analytic code and additional appendices are available on the study's OSF page found here: https://osf.io/s8xpj/?view_only=4598c7667848414f85bb49ea0eb7ceb6.
When available: With publication

Additional Information
Who can access the data: Data is available from the corresponding author upon reasonable request.
Types of analyses: Data is available from the corresponding author upon reasonable request.
Mechanisms of data availability: Data is available from the corresponding author upon reasonable request.
